# Human muscle in gene edited pigs for treatment of volumetric muscle loss

**DOI:** 10.3389/fgene.2022.948496

**Published:** 2022-07-25

**Authors:** Sarah M. Greising, Joshua I. Weiner, Daniel J. Garry, David H. Sachs, Mary G. Garry

**Affiliations:** ^1^ School of Kinesiology, University of Minnesota, Minneapolis, MN, United States; ^2^ Departments of Surgery, Columbia Center for Translations Immunology, College of Physicians and Surgeons, Columbia University, New York, NY, United States; ^3^ Cardiovascular Division, Department of Medicine, University of Minnesota, Minneapolis, MN, United States; ^4^ Stem Cell Institute, University of Minnesota, Minneapolis, MN, United States; ^5^ Lillehei Heart Institute, University of Minnesota, Minneapolis, MN, United States; ^6^ NorthStar Genomics, Eagan, MN, United States; ^7^ Department of Surgery, Massachusetts General Hospital, Boston, MA, United States

**Keywords:** xenotransplantation, skeletal muscle function, human animal chimeras, somatic cell nuclear transfer, blastocyst complementation

## Abstract

Focusing on complex extremity trauma and volumetric muscle loss (VML) injuries, this review highlights: 1) the current pathophysiologic limitations of the injury sequela; 2) the gene editing strategy of the pig as a model that provides a novel treatment approach; 3) the notion that human skeletal muscle derived from gene edited, humanized pigs provides a groundbreaking treatment option; and 4) the impact of this technologic platform and how it will advance to far more multifaceted applications. This review seeks to shed insights on a novel treatment option using gene edited pigs as a platform which is necessary to overcome the clinical challenges and limitations in the field.

## Introduction

Skeletal muscle has a robust ability to regenerate form and function; however, this endogenous process is impaired in rare conditions, such as following complex high-energy orthopaedic traumas (Greising et al., 2020; Eugenis et al., 2021). Volumetric muscle loss (VML) injuries occur following the abrupt and irrecoverable loss of muscle due to trauma or surgical ablation, resulting in irreversible functional impairments (Grogan et al., 2011), and represent a condition whereby the endogenous regenerative capacity of muscle is lost. VML is coupled with clinical outcomes related to long-term dysfunction, reduced mobility and physical activity, and often delayed amputation (Krueger et al., 2012; Stinner, 2016). Clinically, VML is a major problem in military casualties, with ∼77% of all recent U.S. casualties known to occur to the musculoskeletal organs with many having a component of VML (Owens et al., 2007; Owens et al., 2008; Cross et al., 2011) and 8% of those had a VML specific disability rating. There is also significant incidence of VML in non-military traumas and conditions, such as those which are secondary to any of the 150,000 open fractures (Court-Brown et al., 1998), or 30,000 gunshot wounds (Cook et al., 2017), 36,000 chainsaw accidents (Chung and Shauver, 2013), and 13,000 soft-tissue sarcomas (Ferrari et al., 2011) that occur annually. When VML was originally clinically defined in 2011, regenerative medicine approaches were noted as a “*possible therapeutic option*” (Grogan et al., 2011), however over the past decade the field has not yet been able to provide a clinical care option or standard of care to address the loss of skeletal muscle mass and function (Rose et al., 2018; Saunders and Rose, 2021).

The ideal clinical solution for VML will require a large volume of skeletal muscle for transplantation that would deliver all the endogenous aspects of skeletal muscle (e.g., myocytes, satellite cells, basal lamina). However, post-mortem human skeletal is not viable and any autologous tissue transplantation could result in donor-site morbidity which is a reference to the complications and functional restrictions that can occur due to the harvesting of a free flap or graft from a distant site in the same patient. The use of skeletal muscle tissue engineering from myogenic cells and three-dimensional constructs (Iberite et al., 2022) is a promising, yet, still an emerging field and it is unclear if they will be able to produce the volume of tissue needed for successful transplantation.

**FIGURE 1:**
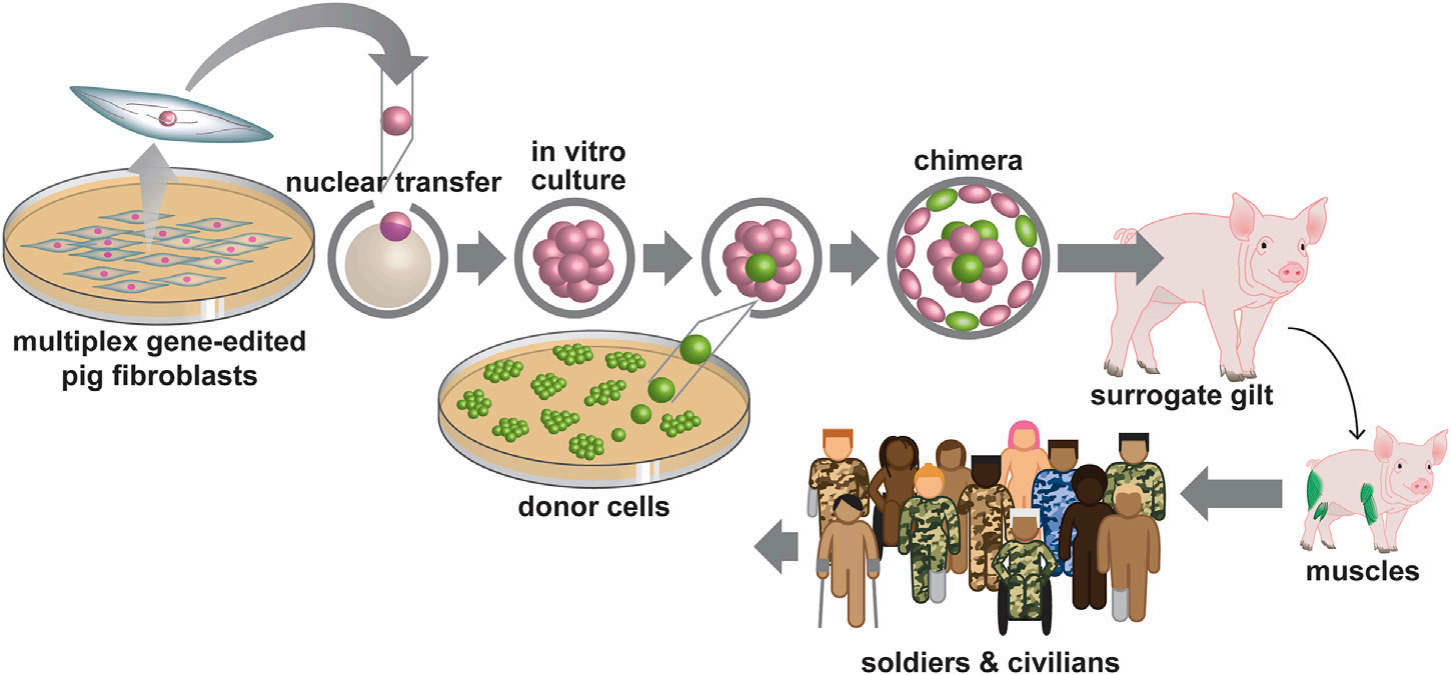
Gene editing, Somatic cell nuclear transfer (SCNT) and blastocyst complementation strategies to produce humanized skeletal muscle. CRISPR based multiplex gene editing is used to delete the porcine skeletal muscle lineage from porcine fibroblasts. Somatic cell nuclear transfer is used to clone the lineage deficient porcine fibroblasts and produce porcine embryos that cannot produce skeletal muscle. Blastocyst complementation is used to rescue the skeletal muscle null phenotype by delivering human cells, to the porcine embryo, capable of producing human skeletal muscle.

The Medical Technology Enterprise Consortium estimates that the military spends in excess of $400,000 in disability costs per patient in addition to lost wages and medical costs. In addition to financial costs, the decreased quality of life and co-morbidities that occur following VML due to decreased muscle mass and altered metabolism, immobility, depression, etc. is significant (Corona et al., 2016; Testa et al., 2021). Thus, there is a major need for novel options. One possibility is to leverage multiple emerging technologies including multiplex CRISPR/Cas9 gene editing, somatic cell nuclear transfer (SCNT), and blastocyst complementation with hiPSCs, in the pig, for a possible multistep solution. By engineering pigs that lack skeletal muscle (Maeng et al., 2021); followed by the subsequent use of blastocyst complementation strategies to rescue the respective gene edited porcine mutant embryos with hiPSCs, the production of humanized skeletal muscle in pigs is feasible (Figure 1).

The production of humanized muscle would allow for the transplantation of mature human muscle which are comprised of intact, innervated and vascularized composites. This would serve as an unlimited tissue source as pigs could be produced based on demand. These pigs can be constructed with human cells from ‘universal donors’ or with personalized human cells derived from the patient. Either strategy could reduce or eliminate the need for immunosuppression in the transplant recipients. It is also possible that differentiated human cells (satellite cells, progenitor cells) could be harvested from these chimeric animals to provide an ample source of cells such that combinatorial therapies (e.g., bulk muscle transplantation together with seeded cells) can be utilized. Foundationally, blastocyst complementation using hiPSCs in the *MYF5/MYOD/MYF6* null porcine embryo to produce humanized skeletal muscle, would serve as an unlimited transplant source for patients with VML injuries as technologies advance. While exact patient information is lacking, a recent case-series of 13 VML injured patients (Dziki et al., 2016) indicates those patients underwent an average of 10 surgeries in attempts to support limb salvage and/or address the VML injury. Humanized skeletal muscle is a possible and promising solution to limit ongoing efforts for limb salvage in these patients.

## Current research gaps: Pathophysiologic impact of volumetric muscle loss injuries

Inherently, the VML injury removes all regenerative components from the injury site, and the injuries are complex and heterogeneous in nature. While much of the current clinical understanding arises from functional data and observations from those injured (Mase et al., 2010; Garg et al., 2015; Dziki et al., 2016); our knowledge of the pathophysiology and mechanisms of failed regeneration has emerged from rodent and porcine models (Corona et al., 2016; Greising et al., 2019) which is paramount to the development of new technologies for transplant sources. Two aspects of the pathophysiology of the muscle at the initially VML injury site are vital to understanding, developing and evaluating treatments; 1) the lost muscle at the primary injury site (i.e., the local muscle environment after injury), failed regeneration, pathological fibrosis (Corona et al., 2020), chronic and heightened inflammation (Larouche et al., 2022), and altered force transmission and muscle architecture (Goldman et al., 2021); and 2) progressive secondary injury to non-injured muscle and systemic insults such as motor neuron axotomy (Corona et al., 2018), denervation (Sorensen et al., 2021), and metabolic inflexibility (Dalske et al., 2021). Multiple potential therapeutic targets exist because the functional deficit is not solely due to the loss of muscle tissue, however, skeletal muscle transplantation near the time of injury could both improve function and prevent the progressive, secondary injury. The field has focused on the development of therapies to promote *de novo* tissue regeneration in the primary injury site and the ability to integrate with the remaining healthy, host musculature. Although necessary, only a subset have focused (and evaluated) on therapies able to contribute to active force production.

No laboratory has produced autologous and fully mature human muscle that can be implanted post-trauma. Currently, surgical reconstruction via free or rotational muscle flap transfer post-VML is an infrequently utilized approach as the use of healthy autologous tissue is limited by donor site morbidity. Certainly, the use of autologous minced muscle grafts has shown promising results functionally and *de novo* muscle tissue regeneration is observed in both small to large animal VML models (Ward et al., 2015; Corona et al., 2017; Aguilar et al., 2018). Moreover, these grafts are minimally manipulated tissues that do not require FDA approval, but their use is limited to small volume injuries. Acellular biological scaffolds, or decellularized extracellular matrices (ECMs), were believed to embody an ideal treatment platform due to their current FDA approval and clinical use for soft tissue repair (e.g., hernia), off-the-shelf availability, and zero autogenous donor tissue burden (Hodde, 2002; Badylak et al., 2016; Yu et al., 2016). To date, however, other pre-clinical reports have not observed appreciable ECM-mediated skeletal muscle regeneration (Corona and Greising, 2016; Greising et al., 2017). Biomaterial developments continue to advance, incorporating growth factors (Baker et al., 2017), supportive cells (Rogers et al., 2021), and novel hydrogel platforms (Basurto et al., 2022; Ziemkiewicz et al., 2022) but advancement into large animals has yet to occur. Recent studies demonstrate that *in vitro* engineered immature skeletal muscle units (SMUs) can survive implantation (autologous tissues) and can integrate with remaining tissue and improve function in rats (VanDusen et al., 2014) and sheep (Rodriguez et al., 2021). Cellular infused bioconstructs show promise in rodents (Machingal et al., 2011; Quarta et al., 2017), yet the harvest and isolation of human muscle stem cells or myoblasts has significant translational limitations and come with inherent complications involving lifelong immunosuppression.

## Potential for future development: Gene editing in large animals and the production of human animal chimeras

Previous research focused on exogenic organ generation demonstrates that deletion of the endogenous organ improves chimerism. For example, a rat pancreas was produced in a mouse with a *Pdx1* deletion by rescuing the mutant mouse by delivery of rat pluripotent stem cells using blastocyst complementation strategies (Kobayashi et al., 2010). Similarly, it has been demonstrated that engineering an organ niche benefits intraspecies chimerism in pigs (Matsunari et al., 2013). The removal of cellular competition in the host has proven to be a beneficial strategy to enhance both interspecies chimerism in rodents and intraspecies chimerism in pigs (Kobayashi et al., 2010; Matsunari et al., 2013).

The feasibility of the production of humanized skeletal muscle in pig relies upon three emerging technologies: CRISPR based gene-editing of porcine fibroblasts, SCNT (cell cloning) of edited porcine fibroblasts, and blastocyst complementation with hiPSCs. It has been demonstrated that the deletion of *MYF5/MYOD/MYF6* using multiplex gene editing results in a porcine embryo that lacks skeletal muscle (Maeng et al., 2021). When blastocyst complementation is used to deliver exogenous porcine stem cells to the skeletal muscle null porcine embryo, the null phenotype is rescued (by exogenous cells which contribute in a near exclusive manner to the skeletal muscle niche) and viable piglets are developed (Maeng et al., 2021). These data support the feasibility of producing intraspecies chimera with this platform technology and, further, support the production of interspecies chimera for the production of pigs with human skeletal muscle. Indeed, human muscle is specified and differentiated following blastocyst complementation of the skeletal muscle null embryo with hiPSCs (Maeng et al., 2021). Studies are underway to advance the gestation times of these humanized porcine embryos to late stage fetal and full-term piglets. It is important to note that interspecies pigs (human:pig) generated with this niche based strategy result in donor cell fidelity to the niche as it has been determined that donor cells do not contribute to other organs, most notably the brain or germ line. A report of the generation of human:pig chimera in a host lacking a niche demonstrated that human cells were distributed throughout the porcine embryo (Wu et al., 2017). The utility of gene-editing is not limited to the development of a vacant niche, *per se*. CRISPR gene-editing is also used to enhance the survival and proliferative capacity of the donor cells (hiPSCs) as has previously been demonstrated (Das et al., 2020; Maeng et al., 2021). Furthermore, gene editing can be used to modify the porcine host cell to be more immunologically receptive to the donor cell. This may be accomplished, for example, by knocking out a lineage such as skeletal muscle to create a competition free zone for the human donor cell (Kobayashi et al., 2010; Wu et al., 2017). Another possibility is to modify Swine Leukocyte Antigens (SLA; Class I and Class II) to create a more hospitable environment for the human cells in the porcine embryo.

Human animal chimeras can be used, not only as a source of humanized muscle for transplantation, but also as humanized models for exploration of human myogenesis, responses to injury and disease, and for toxicology and drug efficacy screens. For example, in the case of VML, injuries (Greising et al., 2017) could be generated in pigs with human skeletal muscle. Reconstructive approaches, immunological barriers, small molecule therapies, and scaffolds and ECMs could be tested for their efficacy in treating human VML. Additionally, using these technologies, pigs can be generated to develop human skeletal muscle with muscular diseases (Duchenne muscular dystrophy, mitochondrial diseases, etc.) by the use of blastocyst complementation with patient derived hiPSCs. Not only would this provide a first of its kind model of human testing, but it would be conducted in the absence of human risk, and it would allow for personalized therapy screening for patients and, thereby, broadly supporting the field of individualized or personalized medicine.

### Tolerance in chimeras produced by gene editing

The successful generation of viable skeletal muscle chimeras (intraspecies chimeras), as described above may have important immunologic implications. Since these chimeric swine are immunocompetent they might be expected to recognize foreign tissue (i.e., donor derived skeletal muscle in this case) and reject it, according to classical immunology pathways. The fact that they do not reject the muscle suggests that a potentially novel mechanism exists for achieving tolerance of allogeneic tissue in this situation. In conventionally understood self-tolerance, host dendritic cells in the thymus display the self-antigens, causing either deletion of developing T cells that bind too-strongly to the antigen-MHC complex or conversion of such potentially-autoreactive cells into regulatory T cells (Treg) (Klein et al., 2014), that can down-regulate T cell responses to the antigens in the periphery, thus avoiding autoimmunity. In the setting of tolerance to allogeneic tissue, such as the muscle in the chimeric pig, these thymic mechanisms would require deletion of T cells and/or generation of Tregs restricted to the donor MHC-antigen complex (Yamamoto et al., 2006). Presumably, either mechanism would require migration of donor antigen presenting cells into the recipient thymus (Remuzzi, 1998). Since the muscle chimeras would be expected to have only donor skeletal muscle, but not donor antigen presenting cells, a different mechanism is required to explain the lack of rejection of the allogeneic muscle.

At present, it is unclear what this novel mechanism may be and several possibilities are under investigation. One possibility may reside with the Autoimmune Regulator (AIRE) mechanism in the thymus, which generates a variety of autologous tissue-restricted antigens by which developing T cells are tolerized to self (Perniola, 2018; Zhao et al., 2018). In these chimeric pigs, the AIRE may behave in a promiscuous manner, allowing the new, donor MHC-antigen of the skeletal muscle to be displayed. A second possibility might involve a novel peripheral mechanism of Treg development. Neither of these possibilities has been described previously. Another possibility that may explain the absence of rejection, but would not really be a mechanism of tolerance, would be anergy, by which the presentation of the donor antigen in a non-inflamed environment can lead to a lack of response to the antigen due to the absence of a co-stimulatory signal. This explanation would not constitute a new mechanism of tolerance, since T cell anergy (or ignorance) can be reversed by exposing the antigen in the presence of an activating “second signal,” such as the production of IL-2 (Nossal, 1993). Exploring these potential mechanisms in skeletal muscle chimeras, using *in vitro* evaluation of donor-specific hyporesponsiveness in mixed lymphocyte reaction as well as *in vivo* challenging of tolerance through transplantation of donor-matched tissue, is the subject of our ongoing work.

## Discussion and summary

The establishment and importance of pig models in biomedical research is clear (Lunney et al., 2021). While the use of the pig for the study of muscular dystrophies (Duque et al., 2015; Stirm et al., 2021) is established, its use for traumatic applications is more recent. The generation of chimeric animals represents an emerging field that holds remarkable potential for the treatment of VML injuries as well as the opportunity for the development of unique clinically based disease models. Therefore, the production of interspecies chimera with human skeletal muscle is an exciting new field which could provide unlimited reconstructive and regenerative material necessary for the treatment of VML injuries.
